# Counselling and knowledge about contraceptive mode of action among married women; a cross-sectional study

**DOI:** 10.1186/1472-6874-6-12

**Published:** 2006-08-06

**Authors:** Pınar Topsever, Müge Filiz, Nihal Aladağ, Ruşen Topallı, Özlem Ciğerli, Süleyman Görpelioğlu

**Affiliations:** 1 Kocaeli University Faculty of Medicine, Department of Family Medicine, Kocaeli, Turkey; 2 Piraziz Primary Health Care Center, Giresun, Turkey

## Abstract

**Background:**

Family planning counselling which covers knowledge transfer about contraceptive mode of action, by enabling informed choice, improves compliance to and efficiency of contraceptive methods.

The objective of this study was to investigate associations between family planning counselling, counsellor and correct knowledge about mode of action of modern contraceptive methods among married women.

**Methods:**

For this cross-sectional study, stratified (according to current modern contraceptive method in use) random sampling was performed from the registries of two primary health care centres. Main outcomes were; prevalence of family planning counselling, professional background of the counsellor and correct knowledge about mode of action. A semi-structured questionnaire developed by the researchers was applied via face-to-face interview. The answers about mode of action were categorized as correct vs. incorrect by consensus rating.

**Results:**

Prevalence of counselling and correct knowledge about mode of action was 49.0% and 39.3%, respectively. Higher educated women were significantly more likely to know the mode of action (*p *< 0.001). Being counselled by a physician (54.1%, n = 120) was not associated with correct knowledge about mode of action (*p *= 0.79). Non-barrier method users were less educated (*p *= 0.001), more often counselled (60.8% vs. 8.0%) and less knowledgeable (*p *< 0.001) about mode of action of their contraceptive method, compared to condom users. Nevertheless, counselled non-barrier method users were significantly more likely to know the correct mode of action of their chosen method (*p *= 0.021) than counselled condom users.

**Conclusion:**

The beneficial effect of counselling on knowledge about mode of action of the more complicated, medical (non-barrier) contraceptive methods suggests that the use of family planning counselling services in primary health care should be promoted; furthermore, counselling strategies and content should be re-structured for better efficacy.

## Background

Modern contraceptive methods, by improving reproductive health, have a positive impact on women's overall health and quality of life [1]. To exert this beneficial effect, correct and consistent use of the contraceptive method chosen by the clients is necessary. This is achieved by promoting correct knowledge via good quality family planning counselling. One of the factors which might affect efficacy of family planning counselling, is the source of information or the counsellor. It has been reported that the most adequate source of information on family planning is the general practitioner [2] but other sources of information on this topic have also been cited [3-7].

The purpose of family planning counselling is to help the client make informed choices about reproductive health and family planning issues. Informed choice, which should-among other topics-cover knowledge transfer about the mode of action of the chosen method [8], has been shown to improve efficiency and compliance to contraceptive method use [9]. Thus, correct knowledge about the mode of action of the method chosen, can be considered an efficacy outcome for family planning counselling. In the current medical literature, there is a considerable lack of studies investigating level of knowledge on particular issues of reproductive health like the correct knowledge about mode of action of the preferred contraceptive method.

The hypothesis of this study was that among women using modern contraceptive methods, counselling and being counselled by a physician were significantly associated with correct knowledge about contraceptive mode of action.

The aim of this study was to investigate factors associated with correct knowledge about contraceptive mode of action among currently married women who are using a modern contraceptive method.

The first objective of this study was to assess the prevalence of being counselled and the proportion of women with correct knowledge on contraceptive mode of action. The second objective was to investigate associations of correct knowledge with counselling and professional background of the counsellor in the studied sample.

## Methods

### Design and setting

The study protocol was approved by the Ethical Committee of Kocaeli University Faculty of Medicine (AEK 238/4, project no: 179, 13.9.2002). A cross-sectional, population-based study was performed between November 2002 and February 2003 in the town of Değirmendere, which is situated 20 km southeast of Kocaeli city center. There are 10 certified reproductive health counsellors working in three primary health care centres, responsible for delivering health care to 7724 women of reproductive age in this town. A probabilistic, stratified random sampling was performed to select the women to be interviewed. The variable used to stratify the sample was the modern contraceptive method in use. As sampling was performed by randomly extracting registry cards, only two of the local primary health care centres could participate in this study because they work on a registry basis.

### Participants

Women of reproductive age, registered at one of the local primary health care centres as users of a modern contraceptive method were eligible for this study. Known pregnancy was an exclusion criterion.

### Sample size

An optimum sample size was calculated to represent 3243 locally registered women using modern contraceptive methods. According to this sample size estimation, 453 women were representative of the total of modern contraceptive users in the town with a confidence interval of 95% (β = 0.9, α = 0.05). The distribution of women of reproductive age according to use of contraception is given in table 1.

**Table 1:** Distribution of women of reproductive age by use of contraception

		**Female population of reproductive age in Değirmendere (n, %)**	**Study population (n, %)**
**Sexually inactive**		2415 (31.3)	-
**Traditional method users**		1343 (17.4)	-
**Pregnant/No contraception**		723 (9.4)	-
**Modern contraceptives**	**Condom**	1429 (18.5)	190 (41.9)
	**Intrauterine device**	1133 (14.6)	161 (35.5)
	**Hormonal method**	368 (4.7)	53 (11.7)
	**Female/male sterilization**	313 (4.7)	49 (10.8)
**Total**		7724 (100)	453 (100)

### Data collection

An easy to use, 10-item questionnaire was developed by the research group and applied by the female researchers via face-to-face interviews to women upon verbal informed consent. Sociodemographic variables and obstetric history were assessed. Furthermore, contraceptive method issues like knowledge on mode of action of the current contraceptive method and the professional background of the counsellor were inquired in a qualitative manner by open-ended questions (What do you know about the contraceptive method you are currently using (its contents, application, mode of action)?, Have you been counselled by medical staff (nurse, midwife or physician) before deciding to use your current contraceptive method?). The results of a pilot survey of 30 interviews were utilized to make changes for better understanding of the questions and to eliminate redundancy. The answers to the question inquiring the mode of action of the contraceptive method in use ("How does your current contraceptive method protect you from becoming pregnant?") were rated by a committee featuring four researchers. The committee produced a consensus rating for each respondent's answer (dichotomized as correct vs. incorrect).

### Statistics

Descriptive results are presented as mean ± standard deviation, percentage, median (range) and mode.

Chi-Square test was used to compare proportions and Spearman correlation coefficient was computed for non-parametric data. Odds ratios (OR) are given with 95% confidence intervals (CI).

Educational level was dichotomised as "low" (below 5 years first level primary school education) and "high" (8 years second level primary school and higher). The counsellors were dichotomised according to profession as physicians and other health care professionals. For subgroup analyses, contraceptive method use was categorized as condom vs. non-barrier methods.

## Results

### General characteristics

A total of 453 women participated in the study. The participants were young (mode = 27 years, range 20 to 54 years), mostly had an educational level of second level primary school and higher and were all currently married. The contraceptive method of preference was the condom (41.9%, n = 190) (Table 1).

General characteristics of the study population are displayed in table 2.

**Table 2:** Sociodemographic and obstetric history of the participants

**Sociodemographics**	
Age (years)	34.4 ± 7.3
Education (low/high)	181/272
**Obstetric history^a^**	
Pregnancy	2 (0–16); 2.6 ± 1.6
Parity	2 (0–9); 2.0 ± 1.1
Living child	2 (0–7); 1.9 ± 0.9

a: median (range); mean ± standard deviation.

In general, the proportion of being counselled was low (49.0%, n = 222), as was correct knowledge about mode of action of the preferred contraceptive method (39.3%, n = 178). Being counselled was negatively correlated with knowing the correct mode of action (r = -0.102, *p *= 0. 031, OR = 0.66, 95% CI: 0.45 to 0.96).

### Education

Seventy-six (41.9%) of low educated women and 146 (53.7%) of high educated women had received counselling for family planning. Higher educational level was correlated with being counselled (r = 0.115, *p *= 0.015).

High educated women were significantly (*p *< 0.001) more likely to know the mode of action of the modern contraceptive in use (OR = 3.02; 95% CI 1.99 to 4.58).

### Counsellor

Over half of the counselled women (n = 120, 54.1%) had been counselled by a physician but this fact was not significantly related to correct knowledge about mode of action of the contraceptive method in use (*p *= 0.79; OR = 1.08; 95% CI 0.62 to 1.88) (Table 3).

**Table 3:** Knowledge of barrier vs. non-barrier contraceptive method users by professional background of the counsellor

		**Counselling +**		**Counselling -**	**Total**
				
		**Physician**	**Other**		
**Barrier (n = 190)**	**Knowledge +**	23 (5.1%)	32 (7.1%)	97 (21.4%)	152 (33.6%)
	**Knowledge -**	5 (1.1 %)	2 (0.4%)	31 (6.8%)	38 (8.4%)
**Non-barrier (n = 263)**	**Knowledge +**	19 (4.2%)	2 (0.4%)	5 (1.1%)	26 (5.7%)
	**Knowledge -**	73 (16.1 %)	66 (14.6%)	98 (21.6%)	237 (52.3%)
**Total**		120 (26.5%)	102 (22.5%)	231 (51.0%)	453 (100%)

### Condom versus non-barrier contraceptive methods

Although, only 32.6% (n = 62) had been counselled, most condom users (80.0%, n = 152) had correct knowledge about its mode of action. The contrary was the fact among non-barrier method users who were more often counselled (60.8%, n = 160) but less likely to know the mode of action of their contraceptive choice (8.0%, n = 21). Condom users were significantly higher educated (*p *= 0.001) and significantly more likely to know the correct mode of action compared to non-barrier method users (OR = 36.46; 95% CI 21.27 to 62.49; *p *< 0.001).

Among non-barrier method users, knowledge about mode of action of their current contraceptive method was low. However, counselled non-barrier method users were significantly more likely to know the correct mode of action of their chosen contraceptive method (OR = 2.9; 95% CI 1.08 to 8.12; *p *= 0.021).

As subgroups contained too few cases for analytic procedures, data on knowledge of condom users versus non-barrier method users by professional background of the counsellor (physician vs. other health care professionals) are descriptively displayed in table 3.

## Discussion

Good reproductive health depends largely on how well informed people are on contraception issues [6]. This study, as an indicator of information level, investigated women's knowledge about mode of action of their current contraceptive method with respect to family planning counselling and counsellor.

### Main results

The present study revealed that in general the proportion of being counselled on family planning, as well as the proportion of correct knowledge about mode of action of the contraceptive method in use was low among currently married women in Degirmendere. The fact that being counselled was negatively correlated with knowing the correct mode of action might be a type I error, which is most likely to be attributable to the predominance of condom users in the population, who were less often counselled but more aware of the mode of action of the condom. Knowledge about mode of action of the current contraceptive method was independent of being counselled by a physician. Overall, non-barrier method users were less educated, more often counselled but less knowledgeable about mode of action of their current contraceptive method compared to condom users.

### Contraceptive choice

In our study, 41.9% of the women of reproductive age in Değirmendere were using a modern contraceptive method (Table 1). This figure is comparable to the average use of modern contraception in currently married Turkish women (42.5%) reported in the TDHS-2003 [10], whereas it is lower than the figure in the province of Kocaeli, which is 53.4% [11]. The condom method was favoured by most of participants of the present study (49.1%) which is considerably higher than the figure for current condom use in Turkey (10.8%) [10] and the rate obtained by Vural et al., who reported a proportion of 16.5% for all barrier methods [11]. This difference can be due to the fact that the present sample was extracted from a town in the province of Kocaeli, where a naval base is situated, which may explain the periodicity of contraceptive need. Another factor might be that many women tend to use the same family planning method as others in their social networks [12]. Even when people are aware of the side effects or failures experienced by other users of a method, sometimes they still tend to prefer it because it is familiar [13].

Our results are also in accordance with the data of a national survey on contraceptive methods in Spain, which revealed the condom to be the preferred method of choice for women of nearly all age and educational groups. This was attributed to a certain degree of distrust in hormonal contraceptive methods and on health policy and organization of family planning services [1].

Tountas et al. have described a prevalent current use of condoms (33.9%) among Greek women, which they attributed to lack of early school based sexual education programmes and/or contraception counselling and the identification of Greek women with passive forms of sexuality [6].

Another reason why the majority of women might prefer the condom could be the fact that contraceptive method use is usually influenced by health care professionals recommending them and physicians have been reported to be more prone to recommend the use of condom, surgical method and the pill [1,4]

### Counselling

In this study, 49.0% of all women using modern contraceptive methods had been counselled; this is higher than the rate of general counselling about modern contraceptive methods (39.9%) in the Eastern Marmara region and also higher than the country average (30.7%) [10]. The relatively higher prevalence of counselled women in Değirmendere can be attributed to the certification programme on "Reproductive Health, Education and Communication" held by the Turkish Ministry of Health between 1997–1999 in the province of Kocaeli, which recommended counselling of all persons presenting with contraceptive need. However, the observed higher prevalence of counselling did not translate into correct knowledge about mode of action. This situation indicates a need to bridge possible communication gaps between counsellor and client by re-tailoring contraceptive information and counselling techniques via rephrasing the content into a more understandable "jargon" for the clients. Further research is needed to assess a possible "information overload" effect in family planning counselling sessions.

### Knowledge (association with education, counselling and counsellor)

Sixty-six percent of counselled women were ignorant about the correct mode of action of their current contraceptive method. These figures indicate that there is much scope for improving the quality of family planning services. Higher educated women were significantly more likely to know the mode of action of the contraceptive method used, which is supported by the results of many other studies on family planning.

In a population-based Turkish survey, 54% of modern contraceptive method users were informed about potential side effects of their method and 44% were told what to do if they experienced any side effects. In the mentioned study, women with second level primary school and higher education were better informed than women with little or no education [10].

Higher educational level and better socio-economical status have been shown to be associated with better knowledge about contraception in a study from Brazil [14]. According to the results of an other study conducted in Bangladesh in 1988, despite knowing about modern methods, most women were not able to identify the advantages or disadvantages of these methods [15].

The participants of the present study were also asked to indicate the source of counselling with special respect to the professional background of the counsellor. All counselled women in the present study had been counselled by health care professionals. Being counselled by a physician did not produce any significant difference in the proportion of women with correct knowledge on mode of action about their contraceptive method. In their study conducted in 1998, Vural et al. have reported a universal wish for information about contraception via health care centers and media communications among females in the province of Kocaeli [11]. In the interval between the study of Vural and colleagues and the present study, the demand in the region for family planning counselling by health care professionals seems to have been met to some extent. The similar effect of counselling, regardless of professional background of the counsellor, on knowledge about mode of action of the current contraceptive method, suggests a beneficial effect of the official certification programme on "Reproductive Health, Education and Communication".

## Conclusion

Counselling for medical contraceptive methods with "more complicated" mode of action than barrier methods should be promoted as it seems to be effective in terms of establishing the premises for the client's informed choice. Especially the fact that contraceptive counselling improved knowledge of less educated women is encouraging. Counsellors should tailor information to the situation, clients and their needs. Further studies are needed to investigate outcomes of new counselling strategies to generate data, which should be used to revise and update health care policies.

## Competing interests

The author(s) declare that they have no competing interests.

## Authors' contributions

All authors read and approved the final manuscript.

P.T. and M.F. study design and coordination, data acquisition, statistical analysis and interpretation of results, drafting the manuscript and revising it critically for important intellectual content. N.A. and Ö.C. data acquisition and management carried out the face to face interviews. R.T. and S.G. interpretation of data and revision of the manuscript for intellectual content. All authors have read and approved the final version of the manuscript.

## Pre-publication history

The pre-publication history for this paper can be accessed here:


